# Is CT bulletproof? On the use of CT for characterization of bullets in forensic radiology

**DOI:** 10.1007/s00414-019-02033-0

**Published:** 2019-03-26

**Authors:** L. E. Paulis, J. Kroll, L. Heijnens, M. Huijnen, R. Gerretsen, W. H. Backes, P. A. M. Hofman

**Affiliations:** 1 Department of Radiology & Nuclear Medicine, Maastricht University Medical Centre, PO Box 5800, 6202AZ Maastricht, The Netherlands; 2 Dutch Police, Region Limburg, PO Box 1230, 6201BE, Maastricht, The Netherlands; 3 Netherlands Forensic Institute, PO Box 24044, 2490AA The Hague, The Netherlands

**Keywords:** Forensic radiology, CT, Bullet, Metal, Material identification, Beam hardening, Photon starvation

## Abstract

**Purpose:**

Forensic investigations could benefit from non-invasive in situ characterization of bullets. Therefore, the use of CT imaging was explored for the analysis of bullet geometry and composition. Bullet visualization with CT is challenging as the metal constituents suffer from excessive X-ray attenuation due to their high atomic number, density, and geometry.

**Methods:**

A metal reference phantom was developed containing small discs of various common metals (aluminum, iron, stainless steel, copper, brass, tungsten, and lead). CT images were acquired with 70–150 kVp and 200–400 mAs and were reconstructed using an extended Hounsfield unit (HU) scale (− 10,240 to + 30,710). For each material, the mean CT number (HU) was measured to construct a metal database. Different bullets (*n* = 11) were scanned in a soft tissue-mimicking phantom. Bullet size and shape were measured, and composition was evaluated by comparison with the metal database. Also, the effect of bullet orientation within the CT scanner was evaluated.

**Results:**

In the reference phantom, metals were classified into three groups according to their atomic number (Z): low (*Z* ≤ 13; HU < 3000), medium (*Z* = 25–30; HU = 13,000–20,000), and high (*Z* ≥ 74; HU > 30,000). External bullet contours could be accurately delineated. Internal interfaces between jacket and core could not be identified. Cross-sectional spatial profile plots of the CT number along bullets’ short axis revealed beam hardening and photon starvation effects that depended on bullet size, shape, and orientation within the CT scanner. Therefore, the CT numbers of bullets were unreliable and could not be used for material characterization by comparison with the reference phantom.

**Conclusion:**

CT-based characterization of bullets was feasible in terms of size and shape but not composition.

## Introduction

In the last decade, medical imaging modalities have emerged as a valuable tool for postmortem examination of victims in shooting incidents by providing non-invasive information prior to and complementary to conventional forensic autopsy [1–3]. In particular, computed tomography (CT) has gained popularity due to its ability to create high-resolution cross-sectional images, in which the location of bullets can be accurately visualized within an unaltered anatomy [4]. Also, gunshot trajectories can be reconstructed, though its accuracy is somewhat dependent on the anatomic region. Bullet identification, however, is currently still performed by ex situ analysis. Therefore, it would be highly desirable to use CT, not only to assess the injuries to the body but also to characterize bullets in situ in terms of geometry and material.

Bullet characterization using CT is not straightforward, as bullets are predominantly composed of metals, with high X-ray attenuation compared with organs and bones [5]. This X-ray attenuation not only is material specific and is caused by inherent properties, such as high atomic number (Z) and electron density (*ρ*_e_), but also critically depends on object size [6]. CT has the unique ability to quantify X-ray attenuation, which is described by the CT number (in Hounsfield units [HU]). The CT number is commonly used to differentiate between various types of organs and tissues [6]. However, with standard reconstruction techniques, the characterization of metals is hampered by the limited range of CT numbers that can be distinguished (− 1000 to + 3095 HU [6]). This can be overcome to some extent by reconstructing images using an extended HU scale (− 10,240 to + 30,710 [6]), which allows quantification of X-ray attenuation values over a much larger range. This has been particularly useful for the identification of heavy and dense materials [7, 8]. However, the strong X-ray attenuation of the bullets’ materials may still result in artifacts that could hamper accurate measurement of bullet geometry and X-ray attenuation [6, 9].

A second difficulty of bullet characterization by CT is the limited spatial resolution in relation to the thickness of the thin metal “jacket”, which with some bullets are coated (typically 300–400 μm) [10, 11]. Due to partial volume effects, it is challenging to visualize this jacket layer and thus to correctly characterize bullets in terms of (1) geometry (internal and external structure) and (2) materials of each component.

The objective of the current study was to perform an in-depth analysis on the potential of clinical CT to characterize bullets. For this purpose, a selection of bullets was scanned in a soft tissue-mimicking phantom. The bullets’ geometry as seen on the CT images was compared with their true specifications. Metal identification was attempted by comparison with a database of metal-specific CT numbers obtained on the same scanner from an in-house developed metal reference phantom.

## Methods

### Metal reference phantom

To enable metal characterization of bullets, a database of metal-specific CT numbers was acquired. For this purpose, a metal reference phantom was developed consisting of the following eight commonly available metals: aluminum 2007 (Al with 3–4% Cu), aluminum 7075 (Al with 5–6% Zn), iron, stainless steel, copper, brass (Cu with 20–40% Zn), tungsten, and lead. Metal properties are given in Table 1. For each metal, a disc (diameter ~ 1 cm; thickness ~ 0.4 cm) was fixed onto a plastic strip (dimensions 0.7 × 3 × 50 cm). The discs were aligned along the long axis of the strip, and centers were separated by ~ 4.5 cm. The phantom was placed in the CT scanner with its long axis along the scanner axis. This setup minimized the interference of X-ray attenuation of discs with one another.

**Table 1:** Metal properties, retrieved from [10, 12]

Metal	Z (−)	Density (kg/m^3^)
Aluminum 2007 (3–4% Cu)	13	2700
Aluminum 7075 (5–6% Zu)	13	2700
Iron	26	7850
Stainless steel	24–28	7900
Copper	29	8930
Brass (60–80% Cu; 20–40% Zn)	29–30	8600
Tungsten	74	19,300
Lead	82	10,950

### Bullets

To examine the ability of CT to characterize bullets in terms of geometry and composition, a selection of bullets was made (*n* = 11) consisting of the ten bullets mostly encountered in gunshot incidents in the Netherlands and a bullet designed specifically for the Dutch Police. Bullet size was measured with a caliper, and bullet composition was retrieved from the vendors’ specifications. An overview of bullet properties is listed in Table 2. As an embedding model, a phantom (dimensions 9 × 14 × 20 cm) was made composed of ballistic gelatin, as a soft tissue-mimicking component. Bullets were manually inserted into the gel at a random orientation. For each bullet, a separate phantom was made. The phantoms were placed in the CT scanner, and each phantom was scanned individually.

**Table 2:** Bullet properties. Bullets are arranged from low to high diameter. FMJ, full metal jacket; RN, round nose; HP, hollow point; WC, wad cutter

Bullet	Type	Jacket material	Core material	Length (mm)	Diameter (mm)	Caliber	Manufacturer
1	FMJ	Cu	Pb	19	6	5.56 × 45mm	Federal
2	RN	–	Pb	12	6	.22 Long Rifle	Remington
3	FMJ	Brass	Pb	12	6	6.35 mm	Fiocchi
4	FMJ	Cu	Pb	14	8	7.62 mm	Prvi Partizan
5	FMJ	Ni/Steel	Pb	15	8	7.65 mm	Sellier & Bellot
6	FMJ	Cu/Steel	Steel/Pb	27	8	7.62 × 39 mm	Sellier & Bellot
7	HP/RN	–	Brass/plastic	13	9	9 mm	Dynamit Nobel
8	HP	Cu	Pb	13	9	.357 Magnum	Geco
9	FMJ	Cu	Pb	15	9	9 mm	PMC
10	WC	–	Pb	17	10	.38 Special	Sellier & Bellot
11	FMJ	Cu	Pb	17	11	.45 ACP	Federal

To evaluate the effect of bullet orientation in the CT scanner on material characterization, three bullets (number 2, 6, and 11) were additionally placed in small sample containers filled with gel. The containers were placed in the CT scanner with the bullets’ long axis oriented (1) parallel to the scanner axis, (2) perpendicular to the scanner axis, and (3) diagonal (in between both orientations), respectively.

### Image acquisition

CT images were acquired on a 192-slice dual-source CT scanner (SOMATOM Force, Siemens, Erlangen, Germany), with a pitch of 0.6 and table feed of 34.5 mm/rotation.

For the metal reference phantom, acquisition and reconstruction parameters were varied to determine the optimal settings for scanning metals. Combinations of tube voltages of 70, 80, 100, 120, and 150 kVp and exposures of 200, 300, and 400 mAs were used. Images were reconstructed with a slice thickness of 5 mm and pixel size of 0.20 × 0.20 mm^2^ (matrix 512 × 512) using both a soft tissue (Br40) and a bone (Br59) convolution kernel. CT numbers were exported on extended HU scale.

For bullets, optimal scan parameters (see Results), as obtained from the metal reference phantom scans, were used. Highest tube voltage (150 kVp) and exposure (400 mAs) were used to obtain the highest X-ray energy and flux. Images were reconstructed using the extended HU scale and a soft tissue convolution kernel (Br40), with a pixel size of 0.35 × 0.35 mm^2^ (matrix 512 × 512 pixels) and a slice thickness of 1 mm.

### Image analysis

Image analysis was performed in OsiriX (version 3.8; Pixmeo Sarl, Geneva Switzerland) and ImageJ (version 1.46r, National Institutes of Health, USA) [13, 14].

#### Metal reference phantom

The CT numbers of the metal discs were determined by plotting regions of interest (ROIs) in a short axis cross section in OsiriX. The ROIs (0.35–0.40 cm^2^) were positioned in the center slice through the discs, carefully avoiding the edges to minimize partial volume effects. The mean and standard deviation (sd) of the CT numbers of the ROIs were used to compile the database of metal-specific CT numbers. The effect of acquisition parameters (tube voltage and exposure) on the CT number was tested for statistical significance (*p* < 0.05) with the Friedman test for related samples in IBM SPSS Statistics 23.0 (New York, USA). To obtain insight in the spatial variation of the CT number within the discs, images were exported as DICOM images (matrix 512 × 512, covering 100 × 100 mm^2^). These images were imported into ImageJ, and a spatial profile plot was made of the CT numbers encountered across the discs’ diameter, at the mid-line of the disc.

#### Bullet geometry

Bullet geometry was evaluated in OsiriX. A 3D MPR image was created, and the three perpendicular cross sections were adjusted to display the bullets across their short axis in one plane and across their longitudinal axis in the other two planes. Subsequently, bullet shape was visually assessed and bullet diameter and length were measured at two image reading settings of window width (WW) and window level (WL): (1) full dynamic range (WW = 20,000–40,000 HU; WL = 10,000–15,000 HU) and (2) manually optimized range (WW = 600–1,700 HU; WL = 19,000–30,000 HU).

#### Bullet composition

Cross sections along the bullets’ long axis were exported as DICOM images (matrix 676 × 673, covering 174 × 173 mm^2^) from OsiriX. These images were imported into ImageJ, and a profile plot of the CT numbers across the bullets’ short axis was obtained at the middle of the bullets’ long axis. The bullets’ composition was determined by comparing the CT numbers from the profile plots to the database of metal-specific CT numbers obtained from the metal reference phantom.

To evaluate the effect of bullet orientation in the CT scanner on the reconstructed CT number, an analysis was performed as described above (matrix 676 × 673 pixels, covering 137 × 136 mm^2^).

## Results

### Metal reference phantom

**Fig. 1:**
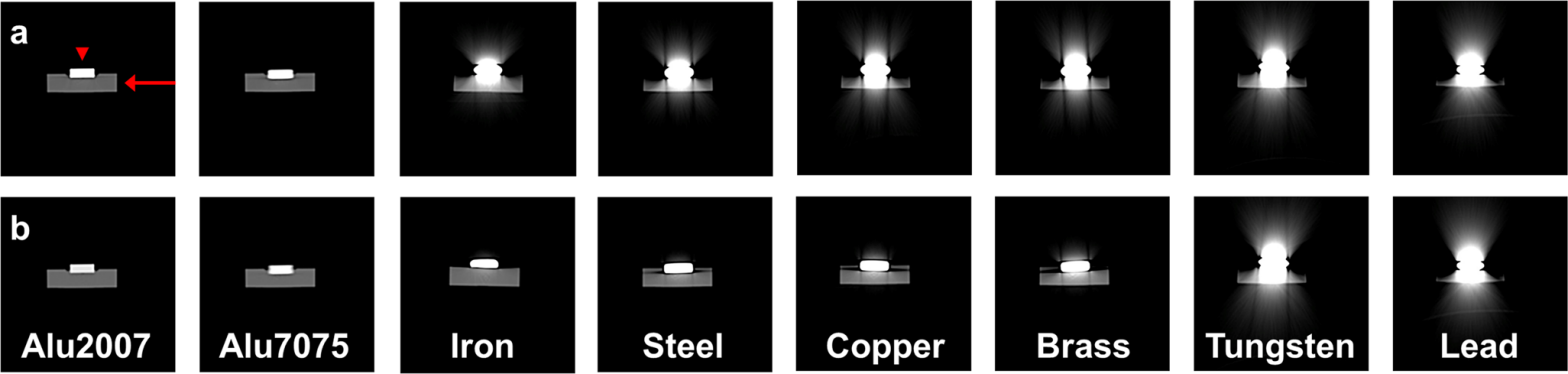
CT images of the metal reference phantom. Short axis cross sections of the individual metal discs at **a** 70 kVp (top row) and **b** 150 kVp (bottom row). Arrow = plastic plate; arrowhead = metal disc. Atomic number (*Z*) increased from left to right

Figure 1 shows cross-sectional CT images of the individual discs in the metal reference phantom, acquired at 70 and 150 kVp. Image artifacts were observed at both tube voltages, but were most pronounced at the lower tube voltage, due to the lower energy of the X-ray beam. At 70 kVp (Fig. 1a), image artifacts were observed for both medium Z-materials (iron, steel, brass, and copper) and high Z-materials (tungsten and lead), whereas at 150 kVp (Fig. 1b) artifacts only were apparent for high Z-materials. Low Z-materials (aluminum) did not display artifacts. These observations indicated that artifact severity increased with Z.

To determine the optimal imaging protocol for metal differentiation, the effect of exposure, tube voltage, and reconstruction convolution kernel was evaluated. Within the range of 200–400 mAs, there was no significant effect on the mean CT number. The effect of tube voltage on mean CT number, though, was statistically significant (*p* < 0.05), with the largest range of CT numbers, and thus the best metal differentiation, observed for 150 kVp. A soft tissue (Br40), rather than a bone (Br59) kernel, slightly increased the CT number range (from maximal 29,029 to 30,317 HU). Based on these results, the database of metal-specific CT numbers was acquired with 400 mAs, 150 kVp, and Br40 kernel.

**Fig. 2:**
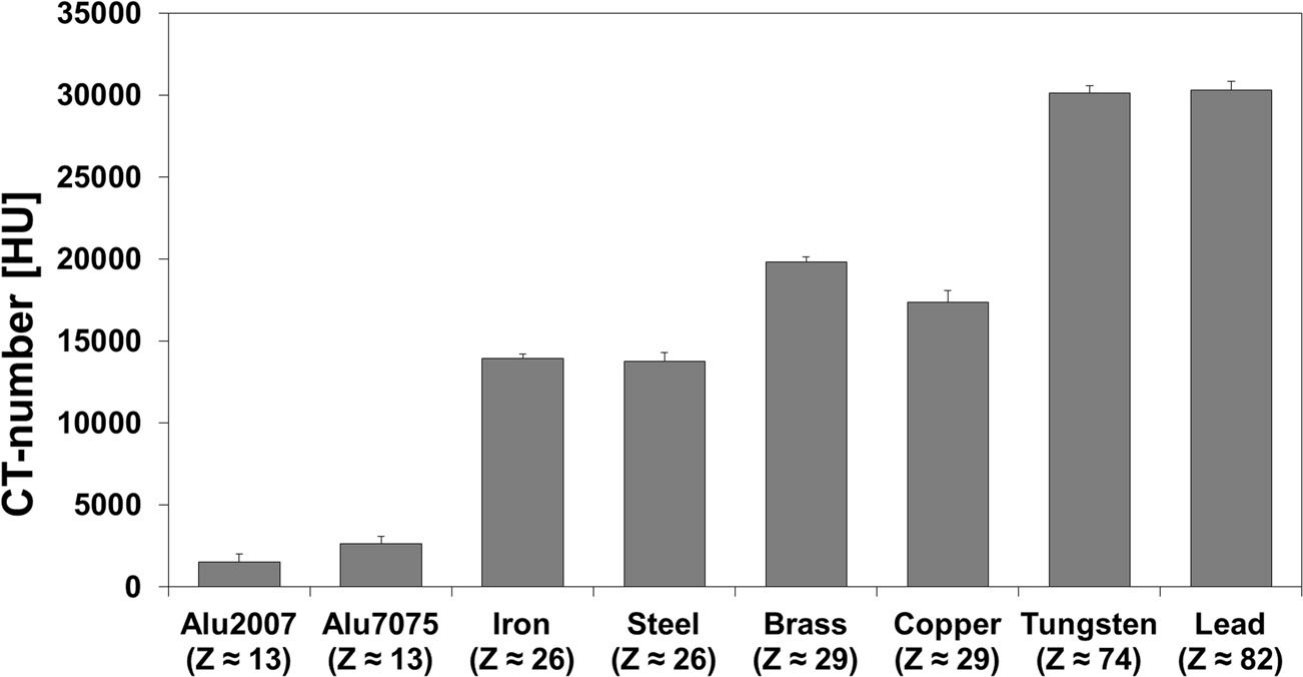
Database of metal-specific CT numbers (mean ± sd) obtained from the metal reference phantom. Data were acquired with 400 mAs and 150 kVp and reconstructed with Br40 kernel

The metal-specific CT number database, described by the mean CT number of each metal, is shown in Fig. 2. An overall trend was observed of increasing CT number with increasing Z, which was indicative of stronger X-ray attenuation. It was not possible to distinguish between metals with comparable Z-numbers. Rather, CT could classify metals into three groups: low Z-number (*Z* ≤ 13; HU < 3,000), medium Z-number (*Z* = 25–30; HU = 13,000–20,000), and high Z-number (Z ≥ 74; HU > 30,000). The gaps in the HU ranges (3,000–13,000 HU and 20,000–30,000 HU) were due to the absence of materials with CT numbers in this range.

**Fig. 3:**
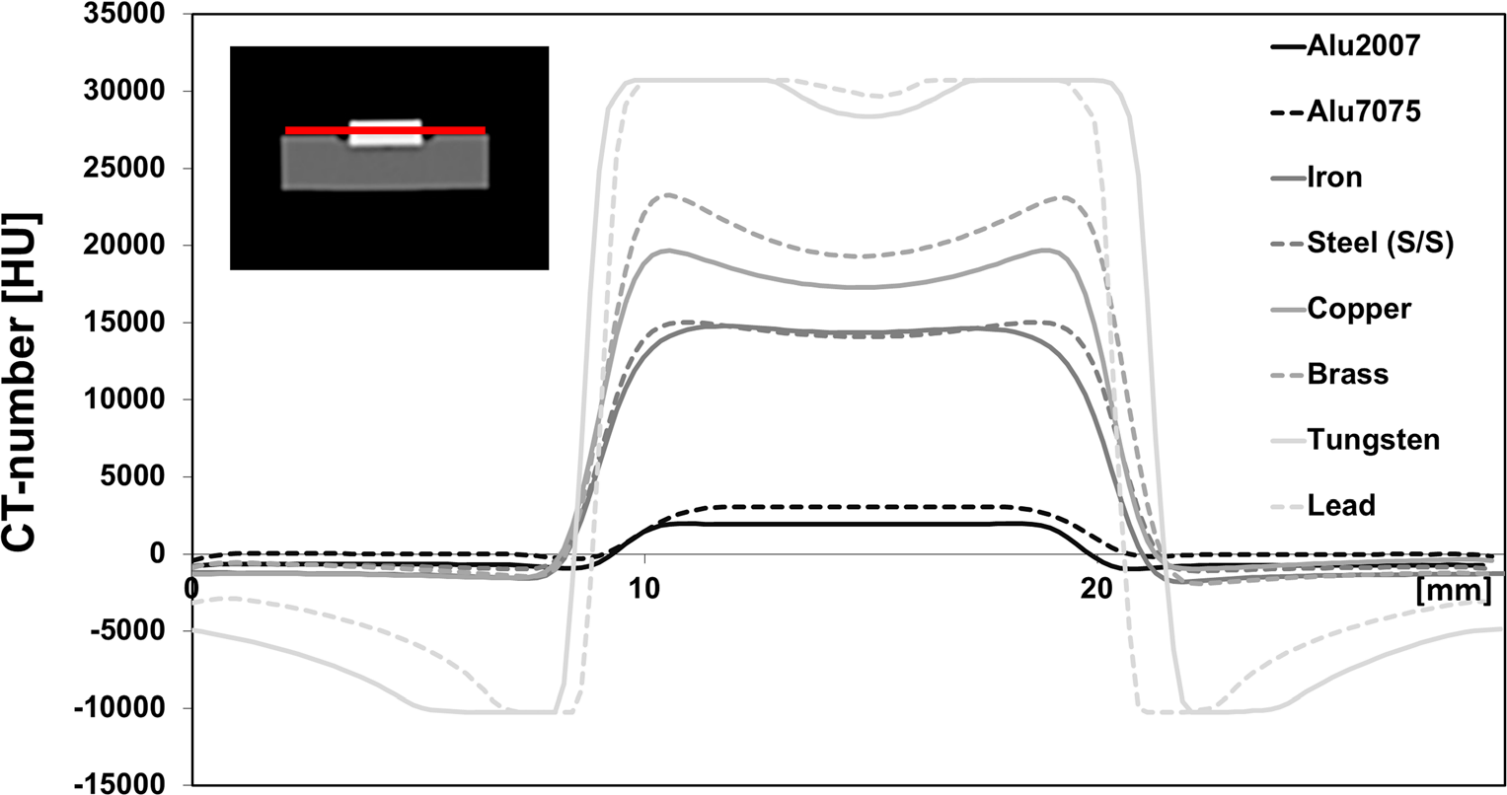
Spatial profile plots of CT numbers across the diameter of the metal discs in the metal reference phantom, as indicated by the red line in the insert. Gibb’s ringing artifacts are observed just outside the discs’ edges (tungsten and lead) and a cupping artifact in the discs’ center (copper, brass, tungsten, and lead). Data were acquired with 400 mAs and 150 kVp and reconstructed with Br40 kernel

To gain more insight in the spatial variation of the CT number within the metal discs, profile plots of the CT numbers were evaluated (Fig. 3). For aluminum, iron, and steel (low and medium Z-metals), the CT number steadily increased from the discs’ periphery towards the center until it reached a maximum plateau value. Interestingly, for copper and brass (medium Z-metals), no plateau was observed, but the CT number reached its maximum value close to the discs’ periphery and slightly decreased towards the center of the discs. This is indicative of a so-called cupping artifact, caused by beam hardening. Beam hardening describes the process by which the mean energy of the X-ray bundle is increased as X-rays travel through the material due to the preferential absorbance of low energy X-rays. As a result, the relative X-ray attenuation is decreased with increasing material thickness, thereby underestimating the total X-ray attenuation in the absence of beam hardening, and thus resulting in an inaccuracy of the CT number. In addition to the cupping artifact, high Z-metals (tungsten and lead) showed a truncation of the CT number at the discs’ edges, indicating that X-ray attenuation was even higher than could be expressed on the extended HU-scale. This was indicative of photon starvation, meaning hardly any X-ray photons were able to traverse the high Z-metals and reach the detector. Most likely, the relatively small numbers of photons that reach the detector were due to scattering (and thus non-linear traversing) in the surrounding (soft) materials of the phantom. Nevertheless, even in the presence of the CT number inaccuracy of some metals, metal categorization into low, medium, and high Z-materials seemed feasible.

### Bullet characterization

#### Bullet geometry

**Fig. 4:**
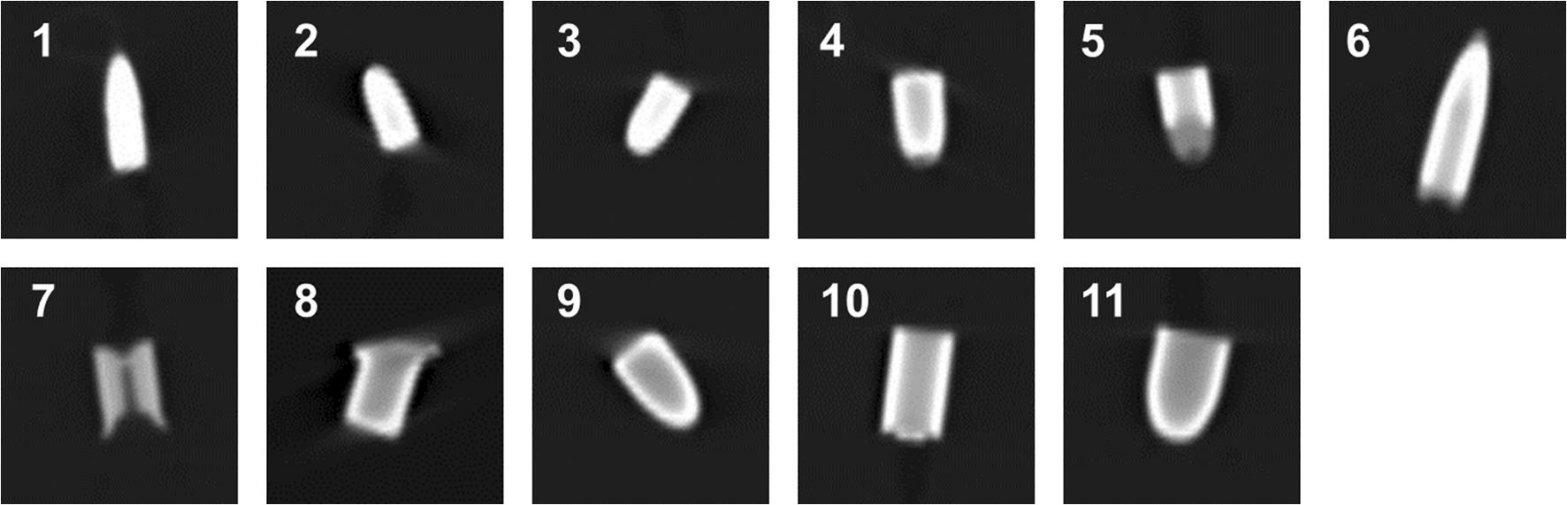
Longitudinal cross sections of bullets in a tissue-mimicking phantom. Images show a selected area of 35 × 35 mm^2^ (138 × 138 pixels). The orientation of the bullets in the images does not represent the orientation in the scanner. Bullet numbering in the image according to Table 2. Data were acquired with 400 mAs and 150 kVp and reconstructed with Br40 kernel

**Fig. 5:**
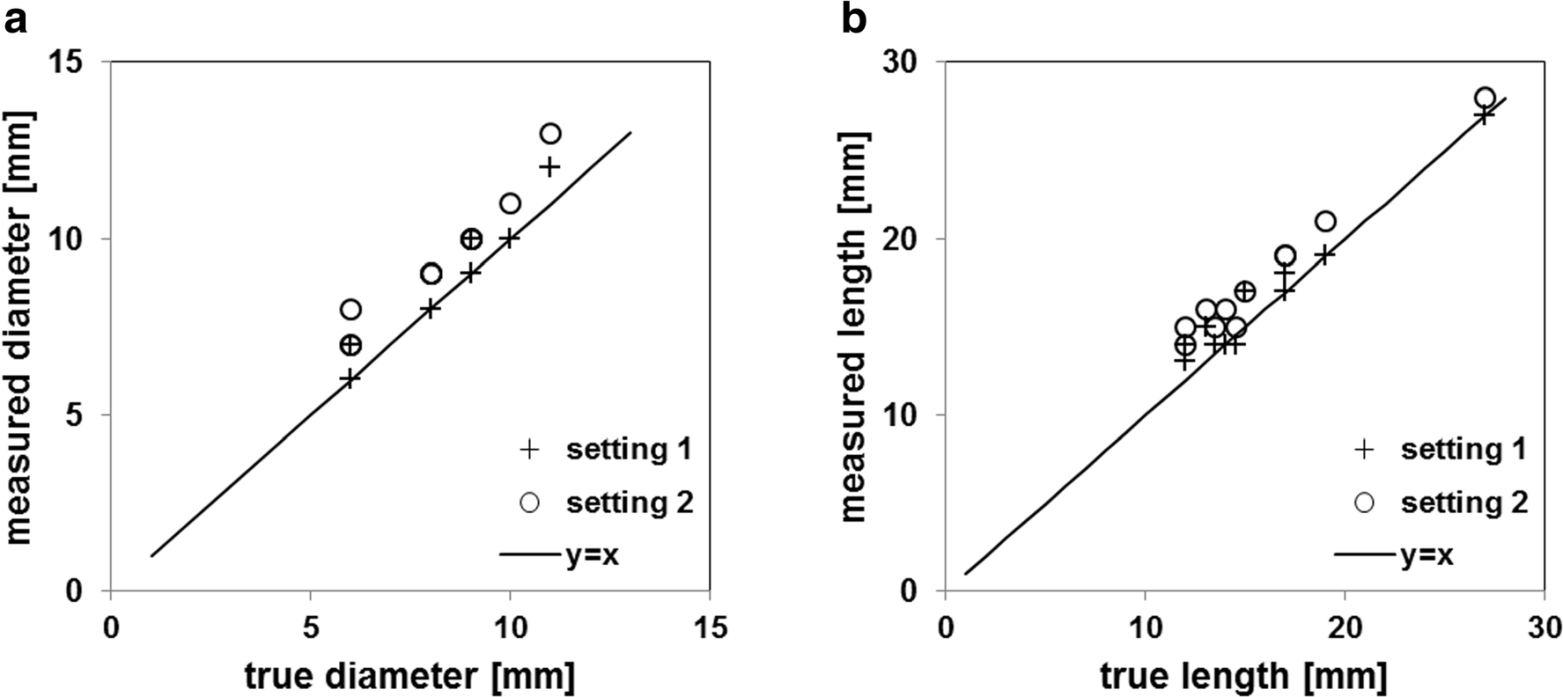
Bullet dimensions as determined from CT images compared with the true dimensions, with **a** diameter and **b** length. Dimensions were analyzed with two image reading (WW/WL) settings. Unity line *y* = *x* is indicative of a perfect match of CT with true dimensions

CT images of the bullets in the tissue-mimicking phantom are shown in Fig. 4. External bullet contours were clearly visualized and not affected by image artifacts, thereby allowing an accurate geometric characterization of bullets. The comparison of bullet size as measured on CT to true bullet size is shown in Fig. 5. CT slightly overestimated both diameter and length, as all measurements exceeded the unity line that represented a perfect match of CT and caliper measurements. Highest accuracy was observed for image reading setting 1 (mean absolute deviation from the true dimensions for diameter and length of 0.5 mm and 0.8 mm, respectively).

Bullet shapes of round-nose and full metal jacket (FMJ) bullets (all except no. 8 and 10) could be visually discerned from the characteristic flat and “deformed” front-end shapes of the wad-cutter (no.10) and hollow-point (no. 8) bullets (Fig. 4). Bullet no. 7, a hollow-point bullet covered with plastic to obtain a round-nose shape, did not appear as a round-nose bullet, since the plastic was not visible with the metal image reading settings.

The jacket layer of FMJ bullets was not visible and therefore its presence or absence could not be determined. This was due to the limited spatial resolution of the CT scanner and the strong X-ray attenuation by the core. Instead, internal “structures” observed on the CT images were merely a deceiving representation of X-ray attenuation artifacts, as described below.

#### Bullet composition

**Fig. 6:**
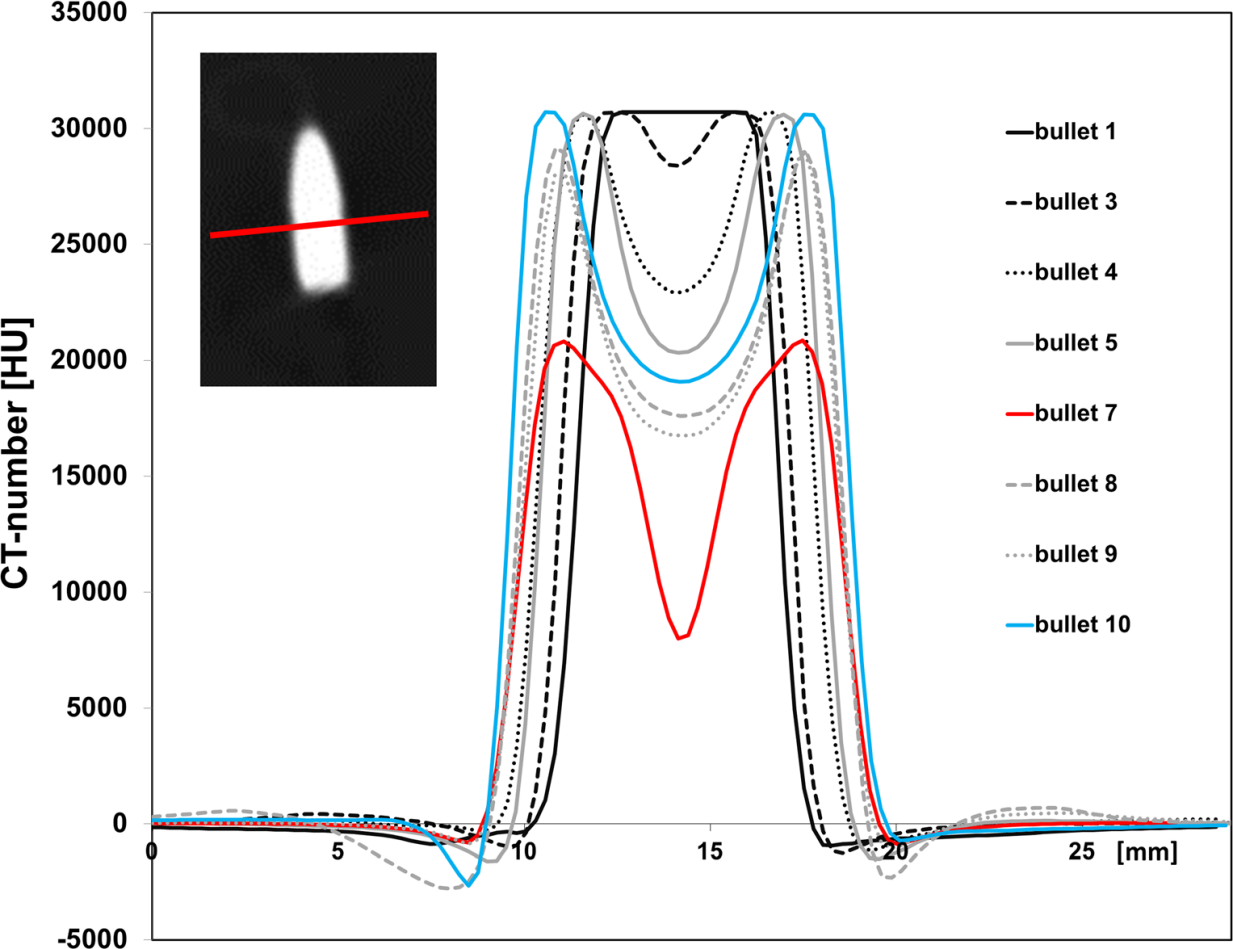
Spatial profile plots of CT numbers across the short axis of the bullets, as indicated by the red line in the insert. Data were acquired with 400 mAs and 150 kVp and reconstructed with Br40 kernel

To evaluate bullet composition, spatial profile plots of the CT number were obtained across the short axis of the bullets in a long axis cross section (Fig. 6). A cupping artifact was observed for all bullets, except the smallest bullet (no. 1). The depth of the cupping artifact increased for larger bullet diameters. Furthermore, it was stronger for the bullets than for the metal reference phantom (Fig. 6 vs. Fig. 3). As a result, the CT number was heavily underestimated in the center part of the bullets and could not be used for accurate material identification. The CT numbers of the bullets’ outer rim (i.e., the peaks in the profile plots) showed no truncation due to photon starvation (except no. 1) and therefore seemed suitable for material characterization.

**Fig. 7:**
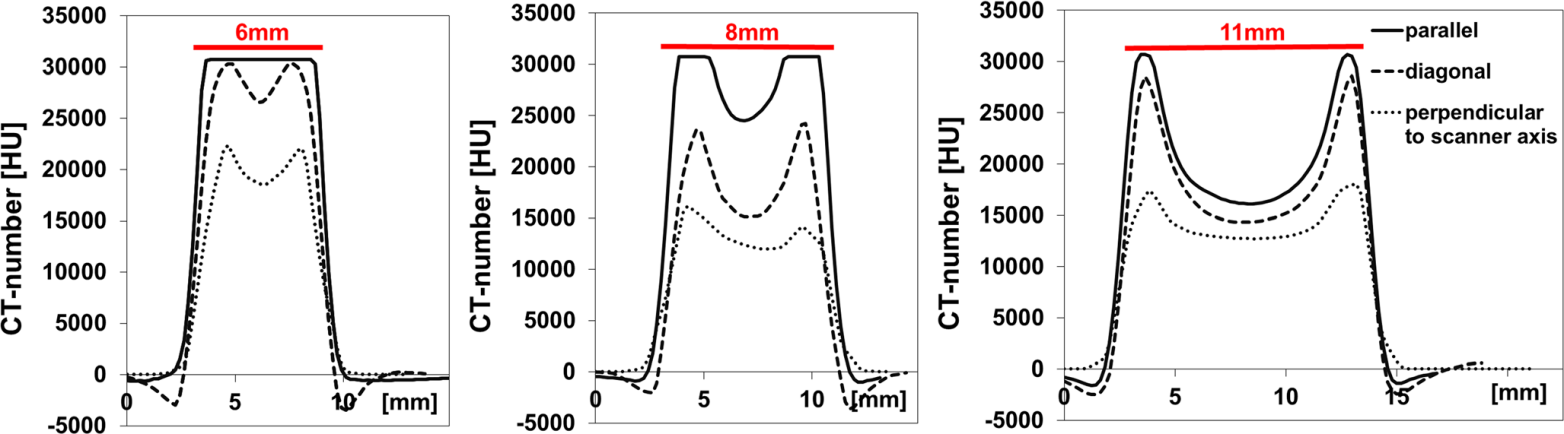
Profile plots of CT numbers across the short axis of **a** bullet no. 2, **b** bullet no. 6, and **c** bullet no. 11, with the bullets’ long axis oriented parallel to scanner axis (solid line), perpendicular to scanner axis (dotted line), or diagonal (in between both directions) (dashed line). The red line indicates the bullets’ diameter. Data were acquired with 400 mAs and 150 kVp and reconstructed with Br40 kernel

To determine whether bullet orientation with respect to the X-ray tube affected the CT numbers, additional CT scans were acquired of a selection of bullets: no. 2 (6 mm diameter, 12 mm length), no. 6 (8 mm diameter, 28 mm length), and no. 11 (11 mm diameter, 17 mm length). As shown in Fig. 7, the profile width (representative of bullet diameter) was not affected by bullet orientation. However, the depth of the cupping artifact and importantly also the peak CT number at the edges depended on orientation: When the bullet’s long axis was aligned perpendicular to the scanner axis, X-rays encountered a thicker material than in parallel orientation and as a result, more X-ray attenuation and ultimately starvation occurred. Therefore, the inaccuracy of the CT number varied with bullet orientation, making not only the CT number of the bullets’ core, but also the outer rim unreliable. Hence, material identification by comparison with the CT numbers of the metal reference phantom was not possible.

## Discussion

Pre-autopsy evaluation of crime scene victims could benefit from the non-invasive characterization of bullet type by CT imaging, in addition to the already used visualization of the bullet in situ trajectory [1–3]. In this work, the capability of CT to assess bullet geometry and material was evaluated. CT characterization of bullets was not trivial due to the excessive X-ray attenuation, which finds its origin in the high atomic number (*Z*), electron density (*ρ*_e_) of metals [5, 6].

As a result of excessive X-ray attenuation, visualization of bullet outer contours was hampered at clinically used image reading settings. Fine-tuning of the image display parameters improved bullet visualization and made it feasible to determine the geometry, in terms of size and shape, on the CT images. Bullet dimensions were slightly (0.5–1 mm) overestimated due to the inherent blurring caused by CT reconstruction algorithms. This overestimation was in agreement with previous studies [15]. Therefore, CT could be used to rapidly provide an estimate of the size and shape of bullets before autopsy. This would also be possible for bullets that have been deformed in the body. Nevertheless, ex situ (physical) measurement of bullet dimensions is still the reference standard for forensic investigations.

Importantly, internal structures, i.e. the presence of a bullet jacket, could not be resolved. This was due to a combination of the limited spatial resolution of the clinical CT scanner and limited contrast within the bullet due to photon starvation. This is in contradiction with a previous study that, to the best of our knowledge, has unintentionally mistaken the manifestation of the cupping artifact, in terms of bright edges and a darker center, for the appearance of jacket and core, respectively [16]. Studies using industrial CT scanners, with higher resolution (13 μm) and tube voltage (320 kVp), were able to distinguish between the jacket and core. They showed that the thickness of the jacket layer was ~ 0.3–0.4 mm, which is comparable to the size of only one pixel (0.35 mm) on our clinical CT images [11, 17]. Unfortunately, such industrial scanners are not widely available and are not suited for whole body applications [11].

In forensic investigations of crime scene victims, CT could be used to provide information on bullet geometry in a non-invasive manner, e.g. when the corpse should stay intact (and thus the bullet cannot be dissected). Also, CT could be used prior to ex situ evaluation as a “quick check” to maintain progress in the criminal investigation (e.g. by excluding certain bullet types or refining search criteria). To determine whether detailed features such as the grooves/lands are discernible on CT images, further research is needed. This pattern of grooves/lands and additional scratches are crucial to determine the exact weapon used in a specific gunshot incident.

Another highly desired application of CT would be to determine the eligibility of corpses for MRI. As previously shown, ferromagnetic bullets will move within soft tissue when brought into the magnetic field of an MRI scanner [18]. This will damage the corpse and possibly alter the bullets’ trajectories, thereby destroying forensic evidence. Therefore, precise detection of ferromagnetic components (iron, nickel, or cobalt) in bullets is necessary. Although the CT number could be used to coarsely categorize metals into low, medium, and high Z-metals, the discriminative power of CT in identifying metals within each class was limited. The “medium Z” class in the metal reference phantom was composed of both ferromagnetic (iron (*Z* = 26), nickel (*Z* = 27), and cobalt (*Z* = 28)) and non-ferromagnetic (copper (*Z* = 29) and zinc (*Z* = 30)) metals, thereby making it impossible to discriminate between these metals. Previous work on this topic should therefore be treated with great caution, and the use of CT for this purpose is not recommended [16]. Also, based on the current findings, it is not possible to determine the core and jacket materials and thus to exclude the presence of ferromagnetic constituents.

This inability of CT to determine the composition of bullets was caused by two effects that perturbed the quantitative nature of the CT signal: beam hardening and photon starvation. As explained previously, beam hardening describes the increase of the mean energy of the X-ray photons as the beam traverses material, whereas photon starvation indicates the point where even the high energy photons are absorbed by the material and only very few photons reach the detector. Reconstruction algorithms have difficulty to cope with these phenomena. They assume that there is a human subject in the scanner and correct for beam hardening as expected in human tissue. Extensive beam hardening as caused by metals cannot be handled correctly and results in underestimation of the CT number. In addition, in conditions of photon starvation, CT numbers must be calculated based on no or only very few linearly traversing original photons, so that the contribution of scattered photons and electric noise becomes dominant, thereby further increasing CT number inaccuracies.

The CT number inaccuracy was found to be dependent on a bullet’s dimensions and its orientation relative to the X-ray beam. During a CT-scan, the X-ray tube rotates around the bullet and for each angular tube position the X-ray attenuation along its path is determined. This attenuation varies with angular tube position, as it is linked to the distance that X-rays travel through the bullet: at the edges of bullets, the attenuation is minor, because for many angular tube positions the distance traversed by X-rays was small. However, for more central parts, the traversed distance was larger, thereby creating more attenuation and thus beam hardening (or even photon starvation). This resulted in unreliability of the CT number. Unfortunately, it is not known to what depth the CT number is reliable.

**Fig. 8:**
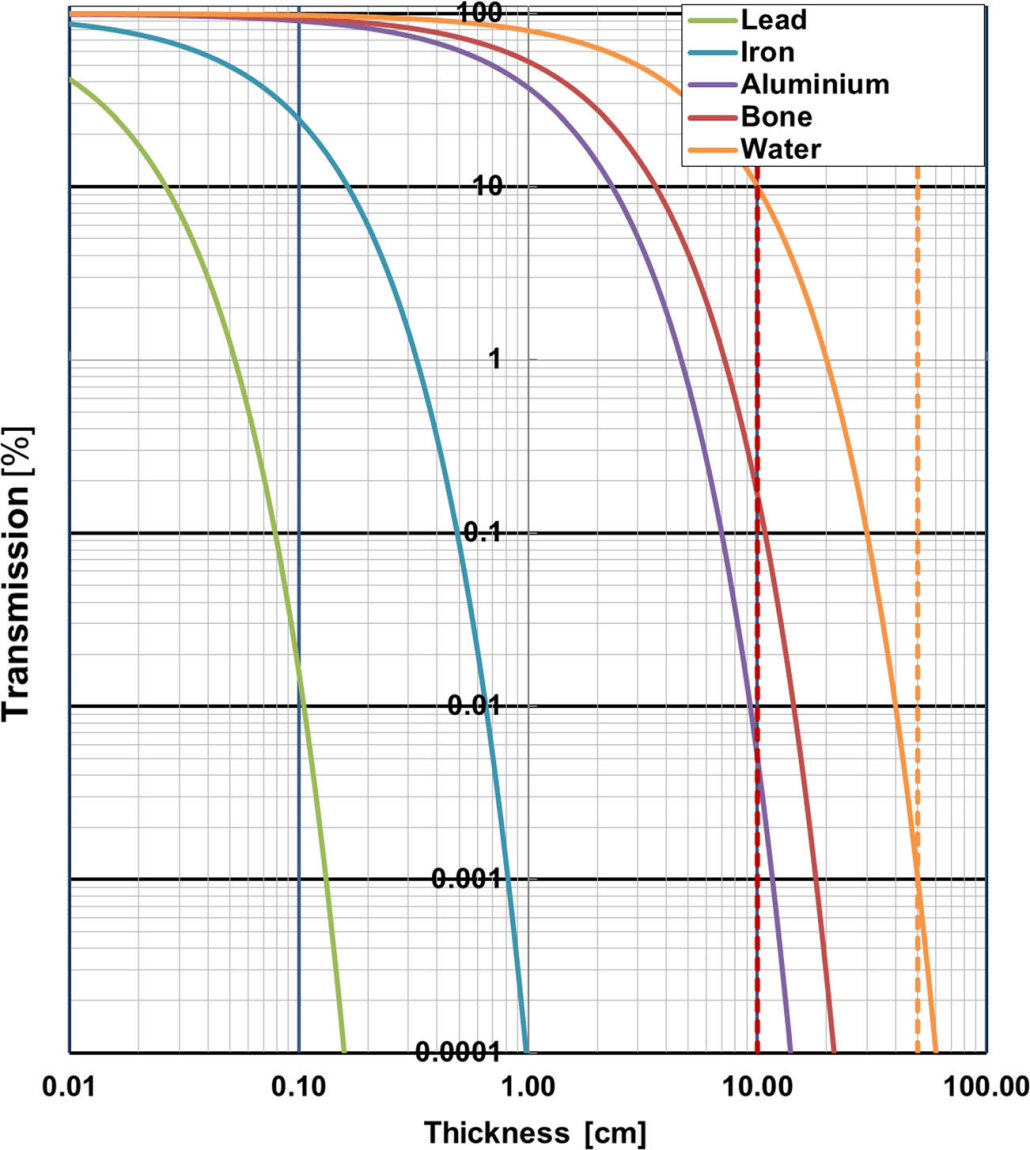
The effect of material thickness on X-ray transmission (50 keV) through various materials. The transmission values range from 100% for air to 0.001% (i.e. 5 decades) for 50 cm of soft tissue equivalent material (water). Dashed red line indicates 10 cm bone; dashed yellow line indicates 50 cm water. Note that for lead, the material thickness cannot be larger than 1.5 mm, in contrast to iron (< 0.8 cm) or aluminum (< 10 cm), for transmission values comparable to a typical body size

For the metal reference phantom, the absolute thickness of the thin discs in the X-ray plane (i.e. perpendicular to the scanner axis) was much smaller than for bullets. As a result, the metal discs suffered less from CT number inaccuracy under identical scan conditions. Indeed, all low and some medium Z materials show little to no artifacts, thus resulting in reliable CT numbers. High Z metals (i.e. tungsten, lead), though, were highly affected by photon starvation and the CT number reached its maximum value. To illustrate this effect, Fig. 8 shows the transmission of X-rays versus the thickness of various metals within the range that can be measured with a clinical CT scanner. For example, for lead, the maximal thickness is 2 mm, thereby indicating that for both metal discs (4 mm thickness) as well as bullets (typically > 5 mm in thickness) there is virtually no transmission and therefore the CT number will be unreliable. However, bullet shrapnel (small fragments) will suffer less from beam hardening and photon starvation artifacts, and might be applicable for material characterization by CT.

Our study had some limitations. First, the tube voltage that is currently available on clinical CT scanners was limited (max 150 kVp). A higher tube voltage (MV-range i.o. kV-range) will result in X-ray photons with a higher energy, which suffer less from photon starvation and thus might enable material characterization [6]. However, with respect to radiation safety, a higher tube voltage is definitely not recommended in living victims. Also in corpses, it remains to be determined whether higher tube voltage (MV-range) can be used without inducing additional radiation damage. This would be highly undesired as it might possibly affect or hamper autopsy findings. Therefore, care should be taken when using high tube voltages for forensic applications.

A second aspect that deserves attention in future studies is the effect of beam hardening and photon starvation on the CT-reading by the forensic radiologist of body parts in close proximity to bullets. This effect can only be determined in crime scene victims, and therefore, was not addressed in the current study.

## Conclusion

To conclude, bullet characterization based on geometric features was feasible using clinical CT. However, recognition of internal structures (jacket vs. core) was not possible. Material characterization suffered from severe beam hardening and photon starvation artifacts, which increased with increasing bullet diameter and also depended on the bullet’s orientation within the scanner. Therefore, comparison of CT numbers to a metal-specific CT number database was not possible.
